# Evaluation of the Digital Alzheimer Center: Testing Usability and Usefulness of an Online Portal for Patients with Dementia and Their Carers

**DOI:** 10.2196/resprot.5040

**Published:** 2016-07-21

**Authors:** Bart Hattink, Rose-Marie Droes, Sietske Sikkes, Ellen Oostra, Afina W Lemstra

**Affiliations:** ^1^ VU University Medical Center Department of Psychiatry Amsterdam Netherlands; ^2^ VU University Medical Center Alzheimer Center; Department of Neurology; Department of Epidemiology & Biostatistics Amsterdam Netherlands; ^3^ VU University Medical Center Alzheimer Center; Department of Neurology Amsterdam Netherlands

**Keywords:** dementia, Alzheimer disease, patient portal, electronic health record, eHealth

## Abstract

**Background:**

Dementia is a progressive and highly disabling neurodegenerative disease that will likely become highly prevalent in the future due to the globally aging population. To improve health care efficiency and quality for dementia care, eHealth could help with, for example, an online portal, such as the Digital Alzheimer Center (DAC) of the Vrije Universiteit Medical Center Amsterdam. It provides up-to-date disease information, peer-to-peer contact, and methods for contacting the hospital and health professionals.

**Objective:**

We aimed to investigate the usability and usefulness of the DAC for patients with dementia and carers to get insight into the feasibility and value of this eHealth app in dementia care and to recommend potential improvements.

**Methods:**

A descriptive study among patients, carers, and health care professionals was performed. Mixed methods were used, consisting of observations (n=10, 4 people with dementia, 6 carers), an online survey (n=287; 88 patients, 199 carers), and semistructured interviews (n=18; 6 patients, 6 carers, 6 health care professionals). During the observations, participants performed a set of five different prescribed tasks on the portal. Speed, number of errors, and navigation were noted. The online survey aimed to assess users’ opinions on the portal’s usability and usefulness. Semistructured interviews were conducted in a subsample of patients, carers, and health care professionals to gain more in-depth information.

**Results:**

In the usability assessment, eight categories of errors were distinguished, of which three were of critical, two of medium, and three of low severity. In the survey, 45% (40/88) of the patients and 53% (105/199) of the carers indicated they used the portal. In all, 33% (12/36) of patients and 61% (62/102) of carers found it easy to learn to work with the portal. Most considered the DAC generally useful: 65% (17/26) of patients and 78% (67/86) of carers found the DAC useful, especially for understanding dementia (patients: 64%, 16/25; carers: 62%, 53/86). In the semistructured interviews, the site was generally rated positively on usability and usefulness and being well designed. People with dementia and carers indicated it helped them to understand and deal with dementia.

**Conclusions:**

To our knowledge, this is the first study investigating the usability and usefulness of an Internet portal especially designed for people with dementia and their carers. An online patient portal could be a useful means to help to support patients and carers in dealing with dementia: the majority of users positively evaluated usability and usefulness of the portal, and appreciated the information on it. However, only a minority of patients found it easy to work with the portal. Good design and frequent usability testing is essential to offer a good online portal.

## Introduction

### Dementia

Neurodegenerative diseases leading to dementia are highly disabling; they are characterized by cognitive decline, gradual loss of daily functioning, and eventually lead to complete dependency on others. Because age is the major risk factor for dementia, the global aging of the population will increase the prevalence of dementia worldwide in the coming years. Additionally, this aging population will lead to a decrease in the available workforce, including professional dementia carers. This will pose a great burden on the care system and on carers. It will also have great economic consequences: in approximately 25 years, dementia is projected to become the disease with the largest economic burden. Worldwide, the economic cost of dementia is estimated to be more than US $600 billion and increasing every year [1]. Therefore, novel solutions to efficiently provide dementia care are urgently needed. In addition to reducing costs, these tools should also improve the quality of life of those with dementia and their carers. One promising tool to deliver efficient care in the future is eHealth: “health services and information delivered or enhanced through the Internet and related technologies” [2].

Limited research into eHealth solutions for people with dementia has been carried out, but initial findings suggest certain applications can help to reduce the limitations that are encountered in daily life [3,4]: it can deliver information and coaching [5,6], it can allow remote consultation [7-14], and its use increases work satisfaction of care staff and improves care relations [15,16]. Additionally, communication tools can promote social contact and GPS- or sensor-based tracking can help to enhance feelings of safety by means of tracking and tracing systems, for example, that can help people with dementia when lost outside of the home [3,17-20].

A promising and increasingly used eHealth solution [21] is an online patient portal: a secure website for patients that offers access to a variety of functions, including secure messaging and protected health information [22]. Portals can offer more personalized health information and social contact. In a 2014 review, Otte-Trojel et al [23] studied 32 papers evaluating patient portals and concluded that these portals can lead to improvements in clinical outcomes, patient behavior, and patient experiences.

### Background

Patient portals are being used for several different (chronic) conditions to offer different services, usually as part of electronic health record (EHR) services. One example is the American MyHealth portal, which uses patient data to generate a personalized health record in which patients can view detailed information about their disease. However, these portals are usually limited in functionality (eg, only offering contact with physicians or only offering access to the health record) and are aimed at the general population of the hospital. They are not optimized for specific patient groups, who may have different needs and wishes. Other portals exist separately from patient records and are often managed by external nonprofit (eg, patient federations) or commercial companies. One example is the patient portal for Dutch cancer patients, kanker.nl, which has more than 15,000 monthly users and is offered by the Dutch Cancer Society. Additionally, there are portals that focus on one specific aspect of support, such as offering information or education (eg, the Skills Training & Re-skilling [STAR] portal for informal and professional carers of people with dementia [24], which offers online e-learning modules), or offer only communication tools, such as the online patient portal nextmd.com offered by Nextgen Healthcare, which only offers contact between patients and their physicians. These are generally offered at a cost, either paid for by health insurance or by the user.

A literature review identified 176 studies that mention portals for viewing EHR data remotely [25]. Although most of these studies were reported to be of low quality, the authors did conclude that users appreciate the added convenience (ie, easy access to information) a patient portal offers. Another review identified 120 articles on patient portals [26]. They found highly variable outcomes: some studies indicated that patients felt that their physicians responded more promptly to their questions than through other means, yet other studies found that users felt an increased workload because of the online portal. However, none of these portals are intended or designed for people with dementia and focus mostly on other chronic diseases, such as diabetes or cancer.

We recently developed an online patient portal, the Digital Alzheimer Center (DAC): the first patient portal on dementia care in the Netherlands. The aim of this portal is to offer comprehensive information on dementia, to enhance social activities, support peer-to-peer contact, and to provide easy access to communicate with health care professionals. A reference group of patients and carers was continuously involved by giving feedback on design and content during periodic focus meetings and usability testing. The DAC was launched in 2012 and has issued more than 1000 accounts since then.

In this study, we aimed to investigate how patients with dementia and their carers value the DAC. We studied this by evaluating two important properties of eHealth and other care innovations that are important for them to succeed: usability and usefulness. *Usability* is defined by the International Standards Organization as “the effectiveness, efficiency, and satisfaction with which specified users can achieve goals in particular environments.” *Usefulness* determines to what extent users judge a website or application to fulfill specific needs.

By evaluating the usability and usefulness this study aimed to provide data on the feasibility and added value of a patient portal in dementia care which can contribute to the existing knowledge on the feasibility and added value of patient portals in dementia care.

## Methods

### Design

To evaluate the usability and usefulness of the DAC, a descriptive, exploratory study was carried out among patients, carers, and health care professionals in which mixed methods were used: observations of patients and carers while they perform prescribed tasks on the DAC; an online survey among patients and carers; and semistructured interviews with patients, carers, and health care professionals.

### Ethics and Informed Consent

This study was approved by the medical ethical committee of the Vrije Universiteit (VU) Medical Center in Amsterdam. For both the observations and interviews, participants received verbal information (by phone) as well as written information (an information letter), after which they were invited to sign a consent form if they were willing to take part in the research. Participants who opened the online survey first were presented with a screen with information about the research after which they could choose to stop or continue with the survey. They could quit the survey at any time without providing a reason.

### Setting and Participant Selection

All participants in the study were clients (patients and carers) and health care professionals of the Alzheimer Center of the VU University Medical Center. The Alzheimer Center is a memory clinic in an academic hospital with a main focus on diagnosing early-onset dementia (dementia with an onset age earlier than 65 years).

Inclusion criteria varied per method. For observations, participants (patients or carers) needed to have participated in at least one DAC workshop (informal workshops organized in the Alzheimer Center, during which participants learn to use the DAC) to ensure that the observed participants had at least some degree of experience with the DAC. This was decided because, for a first exploratory research into the usability of the website, a fully blind “hallway testing” (in which users have never used the site at all) was not warranted yet. Additionally, they had to be physically able to use a computer. Participants were randomly selected from a list of workshop participants of the past four workshops. For the interviews, patients and carers were randomly selected from the list of workshop participants; all professionals that worked with the DAC were approached. An invitation to participate in the survey was sent out to all users registered with an account.

To recruit patients and carers for the observations during prescribed tasks, 10 persons were randomly selected and contacted by a researcher (BH) and asked if they wanted to take part in a usability study on the DAC. For the semistructured interviews, six patients with dementia, six carers, and six professionals participated. Of all 287 users (patients and carers) that started the survey, 40 patients and 105 carers indicated they used the DAC. Of these, 25 patients (63%) and 85 carers (81%) completed the entire survey. Incomplete surveys were also part of the analysis. For an overview of participant flow through the questionnaire, refer to the flowchart in Multimedia Appendix 1. In Table 1, the characteristics of the study participants in each part of the study are presented.

### Overview of the Digital Alzheimer Center

The DAC offers a comprehensive menu containing information on diseases, an overview of appointments and dossiers, community sections, and information on upcoming events and news. The information is written in an accessible fashion and illustrated with animations to clarify pathological processes. Patients and carers can find practical tips on living with the changes that are caused by the disease, financial and legal matters, how to avoid carer stress, and much more. In a specially secured section, patients can email their health care professionals at the Alzheimer Center and view their appointments and medical correspondence. A community hosts a forum for questions and exchanging experiences (eg, a photo and video gallery) and information among patients, carers, and health care professionals. In this forum, users can submit messages on several different subjects (eg, “how to tell family and friends” or “practical tips”) and they can reply to one another’s messages. With the “friends” functionality, users can find others in their area with the same diagnosis and can communicate by a private messaging service. The community section also posts upcoming events and other news from the Alzheimer Center and the national and international Alzheimer community are shared.

**Figure 1:**
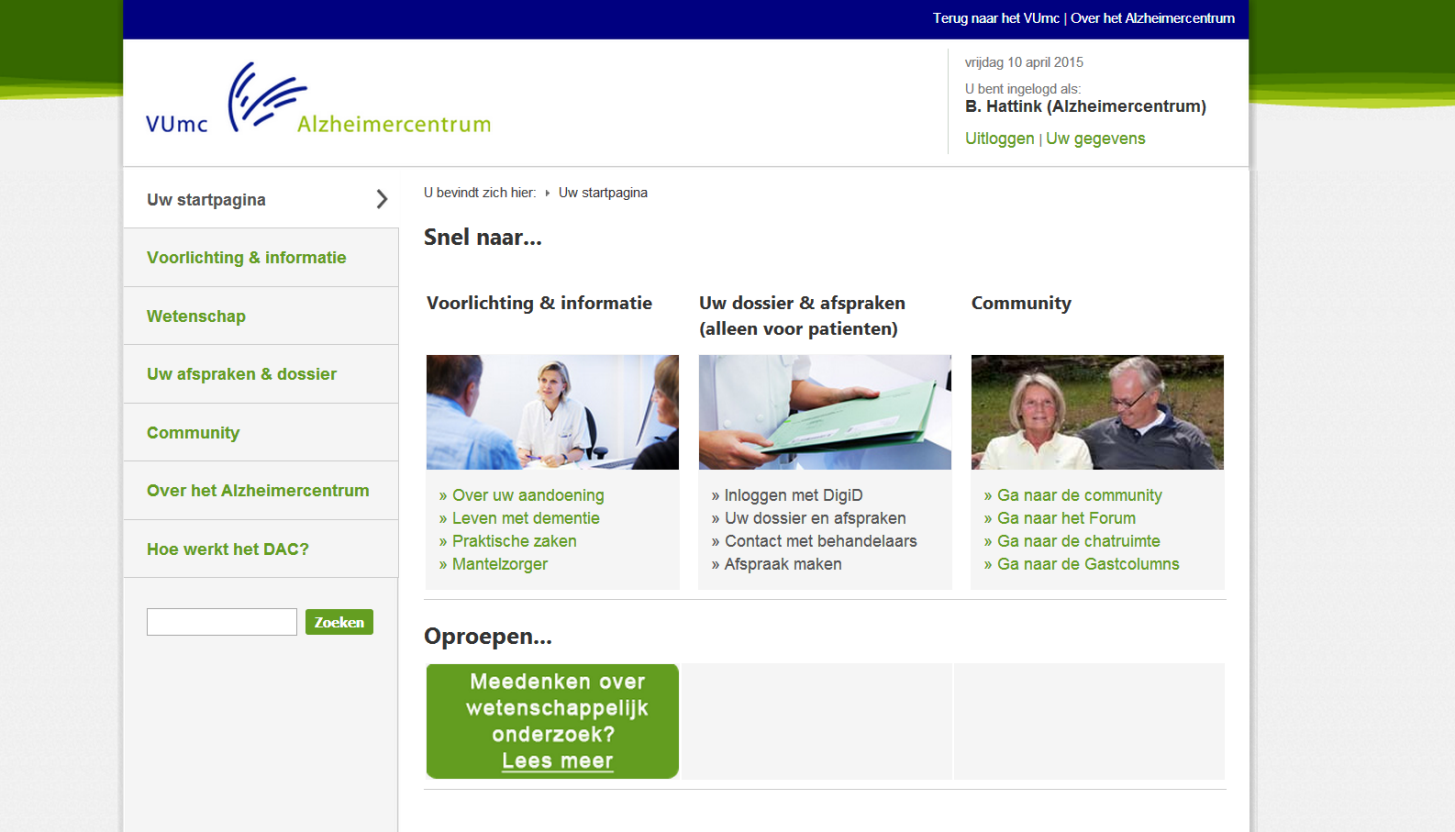
Main (welcome) page of the DAC.

**Figure 2:**
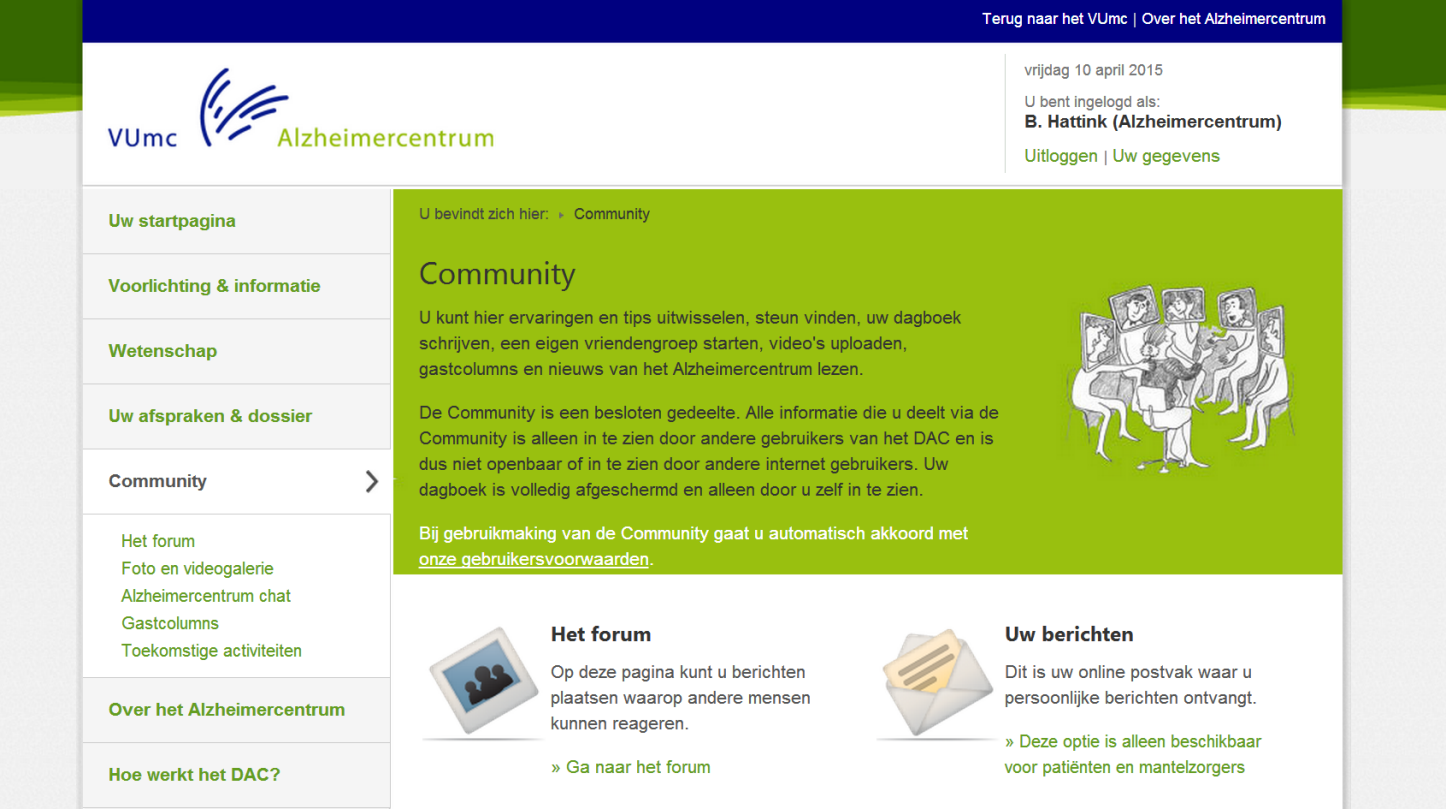
The "community" section.

**Figure 3:**
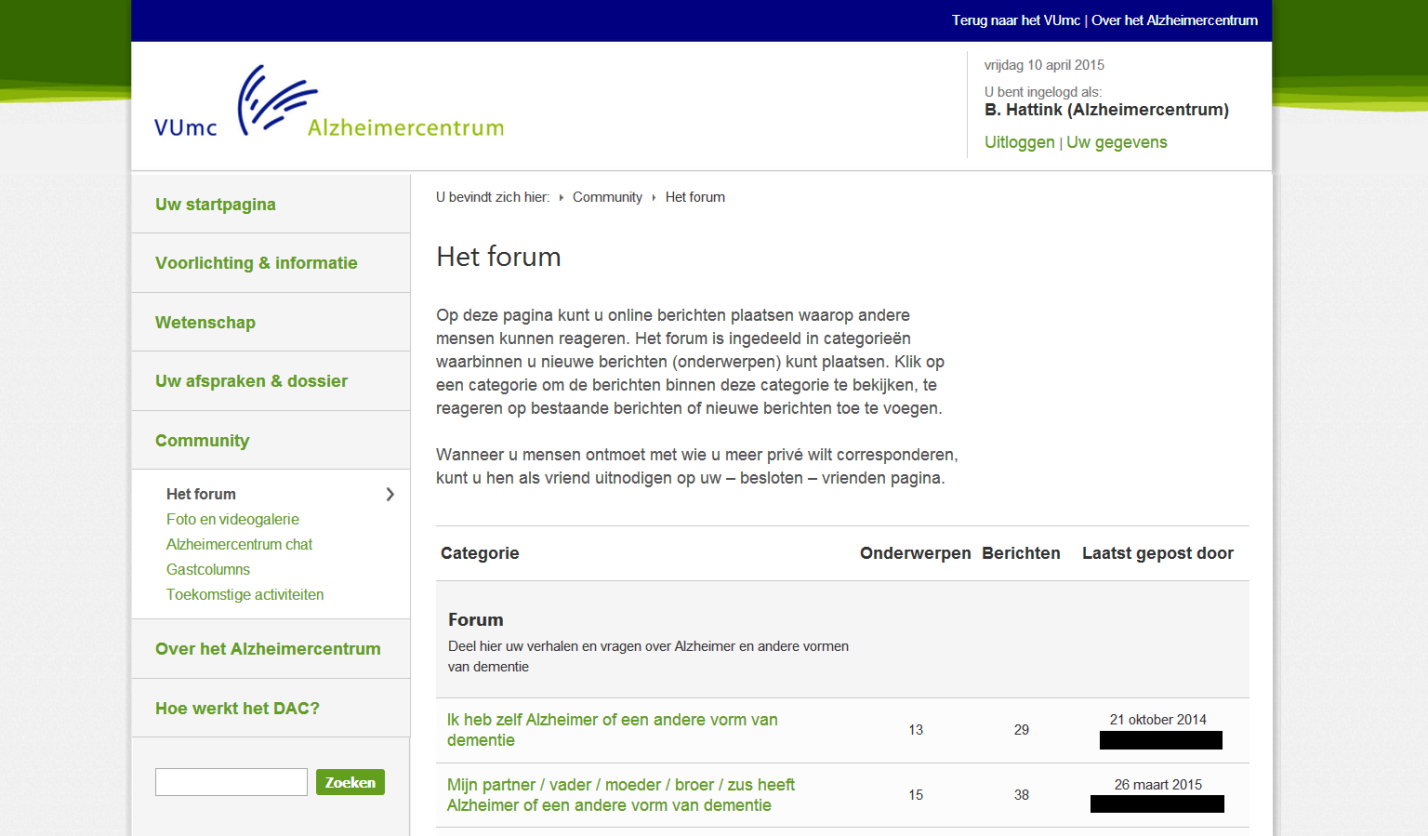
The "forum" functionality.

Detailed in images subsequently is a walkthrough of the DAC in screenshots. The first page, which all users visit after logging in, is a welcome page (Figure 1) where users are presented with an overview of the main functionalities of the DAC. Clicking on one of the options leads further into the website. For example, if they choose “community,” participants are presented with the different functionalities within this section (Figure 2). Within the community, participants can select “forum” (Figure 3) to display all content. Within the “forum” function, participants can select different themes to discuss with others (Figure 3). The DAC can be accessed from anywhere through its URL [27].

**Table 1:** Characteristics of study population.

	Characteristic	Observations during prescribed tasks (n=10)	Online survey (n=287)	Semistructured interviews (n=18)
**Age (years), median (range)**				
	Patient	66.5 (60-79) (n=4)	67 (44-82) (n=88)	71 (61-78) (n=6)
	Carer	72 (58-78) (n=6)	63 (36-82) (n=199)	70 (59-79) (n=6)
	Professional	—	—	44 (29-58) (n=6)
**Patient gender, n (%)**				
	Male	3 (75)	44^a^ (50.0)	5 (83)
	Female	1 (25)	35^a^(39.8)	1 (17)
	Missing	—	9^a^(10.2)	—
**Carer gender, n (%)**				
	Male	3 (50)	41^a^ (20.6)	0 (0)
	Female	3 (50)	80^a^(40.2)	6 (100)
	Missing	—	78^a^(39.2)	—
**Diagnosis patients,** ^b^ **n (%)**				
	Alzheimer disease	2 (50)	109 (54.1)	3 (50)
	FTD	1 (25)	18 (8.9)	
	DLB		17 (8.5)	
	MCI	1 (25)	5 (2.6)	1 (17)
	Other		52 (25.9)	2 (33)
**Patient experience with using computers, n (%)**				
	None	1 (25)	3 (3.4)	1 (17)
	Little	2 (50)	11 (12.5)	3 (50)
	Average	0	34 (38.6)	—
	High	1 (25)	39 (44.4)	2 (33)
	Very high	0	1 (1.1)	0
**Carer experience with using computers, n (%)**				
	None	1 (17)	3 (1.5)	1 (17)
	Little	3 (50)	15 (7.5)	3 (50)
	Average	2 (33)	82 (41.2)	2 (33)
	High	0	78 (39.2)	0
	Very high	0	21 (10.6)	0

^a^Due to an error, gender was not inventoried in the first questionnaire; therefore, these data were collected with a short follow-up questionnaire. Unfortunately, not all participants replied to this questionnaire, which explains the high number of missing values.

^b^DLB: dementia with Lewy bodies; FTD: frontotemporal dementia; MCI: mild cognitive impairment.

### Evaluation Methods

The DAC was evaluated using mixed methods (ie, observations, an anonymous online survey, and semistructured interviews). This evaluation focused on two main outcomes: usability and usefulness.

#### Observations

To assess the usability of the DAC, participants (N=10) were observed while completing a number of predefined tasks on their own computers in their own homes. Several quantifiable measures were recorded during testing. These measures were derived from earlier reports on usability research [28-32]:

1. The type of errors participants made before reaching the end-goal, where “error” was defined by any interaction with the site that did not lead to reaching the goal;

2. The number of errors; and

3. Time on task, the time it took participants to accomplish each task.

The tasks participants were requested to complete involved tasks representative of all functionalities of the site: (1) log in to the DAC, (2) post a message on the forum, (3) find information on driving with dementia, (4) watch a video about Alzheimer disease, and (5) view correspondence with the hospital.

The types of errors noted were errors related to issues with operating hardware, such as the mouse; with operating software, such as the Internet browser; related to navigation of the website; to general understanding of the computer; or other issues that came up. Errors were categorized as low, medium, or critical in severity. For determining severity, the Severity Rating for Usability Problems by Nielsen was used [28]. To determine severity, the number of times “yes” was answered to the following questions was counted and one point was added, making a score of 1 to 4 possible:

1. Does the problem occur frequently or in a critical task?

2. Is the problem difficult to overcome?

3. Is the problem persistent?

Critical errors (score 4) are errors that disrupt website usage enough to prevent actual site usage. Serious errors (score 3) disrupt use and can be frustrating enough to stop users using the site or force them to find workarounds for problems. Medium (score 2) and low (score 1) errors can be bothersome to most users, yet are not likely to directly influence site usage.

#### Online Survey

The online survey contained multiple-choice questions with 4- or 5-point answer scales, regarding background characteristics, such as actual use (eg, “Does one use the DAC?”), and questions on usability and usefulness *.* Usability was divided into three sections: attractiveness (eg, “How do you appreciate the layout of the DAC?”), ease of use (eg, “How easy is it to find the information you need?”), and appreciation of the content (eg, “How understandable are the texts?”). Questions on usefulness concerned the experienced “value” (eg, “Does the DAC help in understanding dementia?”) and “added value” (eg, “Does the DAC offer added value over usual care?”). All questions on the survey are available in Multimedia Appendix 2. This online survey was created in Qualtrics (Qualtrics, Provo, UT, USA). The survey was accessible online for 1 month and contained 82 questions. Several questions were branched and were not shown to all participants (eg, only participants that indicated they did not like the font used on the site were shown the question “What do you dislike about the font?”).

#### Semistructured Interviews

The interviews contained both structured questions and open-ended questions on usability and usefulness of the DAC. The interviews were constructed specifically for this study, using a format of semistructured interviews based on standardized questionnaires, such as the System Usability Scale (SUS) and the User Satisfaction and Ease of use (USE) questionnaires previously developed for evaluation of other technical innovations [18,33] focusing on usability and usefulness. Usability was assessed on two domains: ease of using the site (eg, being able to use the site independently, finding it easy to find information) and attractiveness of the site (eg, appreciation of the layout, colors, font, and images). Two main questions were used to assess the usefulness: added value and areas in which people feel the DAC specifically helps. These questions were either structured with room for comments (eg, “Does the DAC save time?” with options “yes, it saves time; neutral; no, it costs more time”) or open-ended (eg, “What could, in your opinion, be done to make the DAC look more attractive?”). On average, these interviews lasted 21 minutes.

### Procedure

If potential participants consented after initial contact, a researcher (BH) visited them in their own homes, explained the research, and then invited the participants to conduct the prescribed tasks on their own computer, except for two patients who were approached during a workshop and participated directly on a university workstation. A link to the online survey, along with a short explanation of the survey, was included in the monthly DAC newsletter, inviting participants to participate. Patients and carers who participated in the semistructured interviews were recruited among visitors of DAC workshops; professionals were asked to participate via email. Patients and carers were visited in their own homes for the interview by the researcher (BH); professionals were interviewed at their workplace by the researcher (BH).

### Analyses

**Figure 4:**
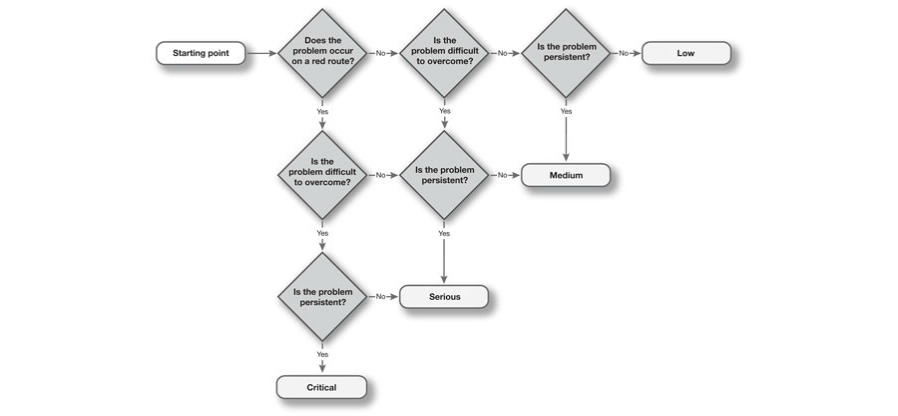
Nielsen's severity rating for errors.

The demographics of the participants in the different study parts and survey data were analyzed with descriptive statistics. Time on task and number of errors made during performing observation tasks were noted. The differences between groups (patients and carers) were analyzed using Mann-Whitney *U* tests (*P*<.05). Errors that occurred were first clustered in themes and subsequently analyzed by assessing the severity of the error. Every newly occurring error was categorized as a new error theme. We kept track of how many other participants made the same error. Subsequently, errors were categorized in the four possible levels of severity: critical, serious, medium, and low (see Figure 4). The online survey data were analyzed per group with descriptive statistics. Differences between the groups were analyzed using the Mann-Whitney *U* test for independent samples (*P*<.05).

Results from the semistructured interviews were analyzed with thematic analysis [19,34]. This was performed both quantitatively by noting the number and the percentage of participants who answered a certain response on structured questions and qualitatively by thematically analyzing answers to open questions or additional comments (eg, by looking for recurring themes in the answers). Some explanatory quotes from participants, representative of the themes we found, were selected to explain the results of the survey.

## Results

### Usability: Prescribed Tasks (Observations)

Time on task of each prescribed task was measured from start to completion. In Table 2, the time on task and the number of errors are presented. A distinction was made between patients and carers. Table 3 shows the different themes of the errors that were made by patients and carers while performing the prescribed tasks and the results of the severity analysis.

**Table 2:** Time on task and number of errors for patients and carers and results of the Mann-Whitney *U* tests.

Task	Time on task (mins), median (range)				Number of errors, median (range)			
	Patients (n=4)	Carer (n=6)	*U*	*P*	Patients (n=4)	Carers (n=6)	*U*	*P*
1. Log in to the DAC	7.5 (0-11)	2.5 (3-10)	9.0	.61	1 (0-5)	2 (0-5)	9.0	.61
2. Post on the forum	10.5 (2-18)	5.5 (2-18)	12.0	>.99	0 (0-5)	0 (0-3)	7.0	.35
3. Find information on driving	8.5 (2-18)	3 (0-17)	11.5	.91	1 (0-7)	4 (0-2)	6.0	.26
4. Watch a video on Alzheimer disease	5 (1-10)	5.5 (1-9)	8.0	.48	0 (0-19)	12 (1-9)	6.0	.26
5. View correspondence	4 (3-10)	3 (3-4)	1.5	.57	0 (0-22)	0.5 (1-1)	5.5	.38

**Table 3:** Thematic overview and severity of errors made by patients with dementia (n=4) and carers (n=6), coded for severity.

Theme of error	Severity
Unable to log in to DAC	High
Entering wrong URL / not finding right page	High
Visiting sites external of DAC	High
Reading the sidebar as part of the other text	Medium
Following wrong links (that do not lead to required data)	Medium
Unnecessary clicking	Low
Unnecessary use of the “back” button	Low
Clicking nonlinks	Low

The biggest difference in time to completion was found, both for patients and carers, within task 2 (post a message to the forum), for which there was a 16-minute difference between the slowest and fastest performance. However, this task had a low variance in errors, with a minimum of zero and a maximum of five errors. The greatest variance in number of errors as well as the greatest difference in performance between patients and carers was found in task 5 (view correspondence with the Alzheimer Center), where the best performing participant did not make errors at all and the participant who had the most difficulty with the task (a person with dementia) made 22 errors before arriving at the right solution.

Patients and carers differed in the number of errors made in three of the five tasks: post a message on the forum, finding information on driving, and watching a video on Alzheimer disease. The tasks appeared more difficult for patients because they made more errors and took longer to complete the tasks. However, from the Mann-Whitney *U* tests, these differences between patients and carers did not appear to be statistically significant.

Both patients and carers made the same categories of errors (refer to Table 3), except for not understanding the sidebar, which only occurred in patients with dementia. All problem themes observed were analyzed by using the severity framework of Nielsen [28].

### Usability: Survey and Semistructured Interviews

#### Layout

The results of the survey show that the design of the site was appreciated by a small majority: 19 of 36 (53%) patients and 60 of 98 (61%) carers indicated that the layout was clear. They appreciated that it “looks very calm, there’s no clutter [distracting elements] on the screen” (indicated by a carer). The font used in the design of the website was appreciated positively: only 2 of 98 (2%) carers did not like the font.

#### Content

The content of the DAC was rated understandable and clearly written by both carers (79/96, 82%) and patients (27/35, 77%) in the survey. The information was regarded “very well and comprehensively written” by all interviewed participants.

#### Ease of Use

Survey participants valued the site mostly positively with regard to ease of use (general use and navigation). In all, 50.0% of carers (52/104) found it easy to use, 36.5% (38/104) were neutral on this subject, and 13.4% (14/104) found the site difficult to use. For patients, 42% (15/36) rated the site as easy to use, 50% as neutral (18/36), and 8% (3/36) as hard to use. All but one patient of those interviewed thought that they would be able to learn to use the site.

### Usefulness

#### Added Value

Overall, 17 of 26 (65%) patients and 67 of 86 (78%) carers indicated in the survey that the DAC was “very useful” or “useful” and both indicated it had an added value over the regular care offered by the center. Interviewed participants specified that it was “very helpful—it really helps me in staying at home by myself” and that it “should certainly be continued in the future.” One professional commented that it was “not yet useful enough,” although they later indicated that they expected this would change by “adding more personalization [options].” A majority of users, 17 of 26 (65%) patients and 57 of 86 (66%) carers, would recommend the DAC to others: “It is certainly something you need in this day and age.”

#### Understanding of and Dealing with Dementia

Participants indicated that the DAC was especially useful to them for understanding dementia and for dealing with dementia. In all, 53 of 86 (62%) carers and 16 of 25 (64%) patients who responded to this question indicated it was helpful for understanding dementia: “you can find all the information you might need” and “you can easily show this information to others.”’ In addition, 40 of 86 (47%) carers and 11 of 25 (44%) patients found the DAC useful for dealing with dementia. The availability of the information was appreciated: “you can check this information anytime, even in the middle of the night.”

#### Usage

In the survey, 145 of the total 282 (51.4%) participants indicated that they had used the DAC at least twice. Of these 145 users, 40 (27.5%) were patients and 105 (72.4%) were carers. Participants in the semistructured interviews also indicated they regularly used the DAC; all but two indicated they did not use it. One interviewed patient specifically stated that he used the DAC “several times a week.”

In Table 4, survey data are presented on the use of features of the DAC. It shows that both patients and carers make (more or less) use of all different parts of the site. Most used by patients are the information on the disease and the Alzheimer Center function. Most used by carers is information on the Alzheimer Center and information for carers.

**Table 4:** Use of functions: numbers (and percentages) of patients and carers that used a specific function.

Function		Patients, n (%) (n=25)	Carers, n (%) (n=85)
**Information**			
	Disease	12 (48)	14 (16)
	Informal carers	1 (4)	20 (24)
	About center	10 (40)	44 (52)
**Community**			
	Forum	7 (28)	13 (15)
	Friends	4 (16)	3 (3)
	Chat	3 (12)	2 (2)
**Contact**			
	E-consult	6 (24)	17 (20)
	Correspondence	2 (8)	9 (11)

### Non-users

When participants indicated in the survey that they did not use the DAC, they were asked why they did not use it. Their answers were grouped into themes. The main reasons they indicated for not using the DAC are presented in Table 5.

**Table 5:** Reasons for not using the DAC.

Reason	Carer	Patients
1	No need (n=31)	Miscellaneous (eg, “I don’t want anything to do with it”) (n=12)
2	Technical or computer issues (n=23)	No need (n=9)
3	Miscellaneous (eg, “I don’t like the Internet”) (n=18)	Unfamiliar with DAC (n=8)
4	No time (n=13)	Too hard to use (n=7)
5	Unfamiliar with DAC (n=8)	No time (n=4)
6	—	Technical or computer issues (n=4)

## Discussion

We found that, in general, patients with dementia, carers, and health care professionals who use the patient portal rate it positively with regard to usability, and consider it to be a useful addition to existing care that helps them to deal with dementia, among other things. Results for this study show that an Internet portal is a feasible means of offering support to people with dementia and carers. Both patients and carers indicate they appreciate such a portal positively. Although some had trouble in using the site or in learning to operate it, only a small percentage of users responded negatively to the patient portal as a means of offering support. The information sections especially appear to be well used and are indicated to be experienced as supportive.

Nonetheless, we did also find some usability issues. The most notable issues are those functionalities for which severe errors were found during usability testing: the log-in screen, the process of finding the right URL, and the confusion of leaving the DAC for a different linked site. A positive note is that these are all areas related to reaching and accessing the site, and have nothing to do with the actual (functioning of the) site itself. We did find that patients with dementia and carers largely make the same kinds of mistakes, which means this is likely to be related to familiarity with using computers and websites.

Findings from this study are in line with previous research in this area [4,17,35-41]. We found that older users and users with dementia are able and willing to utilize Internet-based resources and that at least some of them are capable of using the technology involved. Research by Ellis and Kurniawan [35] showed that older users consider the Internet a useful tool for finding information and that they were able to access websites on computers with relatively few problems. Research into website usability among people with dementia found that they prefer websites that have little cognitive load (ie, “the amount of mental processing power needed to use the site” [36]) and that minimize the amount of clutter [35] and other distractions on the screen, such as on-screen animations and advertisements [37]. Besides making sites harder to use, earlier research states that cognitive load and clutter may cause “knock-off effects,” causing people to require so much cognitive effort for processing site usage that they cannot effectively process or engage with the material on the site [4,38]. The current research confirms these findings. In the observations, we found that users occasionally had trouble finding the correct links and, in the interviews, users mentioned that they appreciated how few distracting elements there were.

Additionally, decreased motor skills and slower movements that occur in older age could affect the use of scroll bars or links and buttons [38]. This was found in website use as well as in usability studies of other technology such as mobile phones [39]; when observing the difference in usability of mobile phones between older and younger users, it was found that older users could use mobile phones but had significantly more difficulty with more complex mobile phones [39].

Research by Chadwick-Dias et al [40] tested several enhancements to a website to make it more usable and found that clearer wording of links, more consistent visual identification of links, and the use of simpler terminology significantly improved performance on a website. These findings concur with the findings of our research: our users had some trouble identifying links. The simple and understandable language used on the DAC was appreciated by the participants. When research participants were offered two different versions of a website with the same information but with different layouts (one complex with lots of information on screen, one simple version with little information displayed at once), participants made fewer errors on the website with less complex screens [36,41]. Participants also rated the less complex site as more attractive and better to use [36].

This study highlights the importance of iterative development, in which user needs are assessed at the start, and the target audience participates throughout the process [19]. Design choices such as clear font, calm backgrounds, and contrasting colors are important to ensure optimal usability. These design considerations were all applied in development of the DAC and the majority of users evaluated these aspects positively or very positively. This is in line with earlier research into typography for websites [42,43]: when learning to work with computers, a 12- to 14 point sans serif typeface is best appreciated by older users and improves their reading performance on the screen. They also found that it is important to use contrasting colors (preferably black text on a white background) to ensure readability.

Based on findings from our study and on earlier research, we made several practical suggestions for good website design, which can be useful for others intending to design an online portal for people with dementia and their carers. It is recommended to resolve usability issues as soon as possible. For example, problems with finding the URL could be alleviated by adding redirects on more URLs (eg, variations and typos of the current URL www.digitaalalzheimercentrum.nl). Problems with leaving the site for another site could be solved, for example, by a warning page that lets users know they are about to leave the DAC and will be presented with another site with a different layout than the DAC. The different layouts of other sites, which are generally not specifically designed for older users or users with dementia, make them very confusing. Another critically severe error found during the observations was that participants had trouble logging in to the DAC. Because this is a very critical step—it being the first contact with the portal—it is highly relevant to find ways to fix this (eg, by allowing log-in information to be saved or by considering other means of logging in).

We did not find any indication of harmful effects of the website. Some participants indicated that they did not use the DAC because they did not want to be confronted with all the information about their prognosis (several of the nonusers presented in Table 5 mentioned this when they indicated “no need” as reason for nonuse). However, because use of the DAC is voluntary and not required for any services at the Alzheimer Center, there is no need for them to be confronted with this.

### Limitations of the Study

The survey was sent out to all patients registered with an account for the DAC. Even though the response rate was relatively high for online surveys (39%; research generally reports rates from 5% to 40% for online surveys), there is no telling if the group that responded was representative of the population. It is possible that those positive toward the DAC were overrepresented in the group that responded.

To ensure that participants were familiar with the DAC, those taking part in the observations were selected from people who had participated in a workshop in which they learned to use the website. A group of participants that uses the site for the first time without any explanation may encounter different problems. It should be noted, however, that the latter group is not the target group of the current portal: it was specifically intended and designed for people with dementia who were patients of the Alzheimer Center. They were all invited to join a workshop to learn to use the DAC.

To further elaborate on the outcomes of this study in the future, answers to both the survey and the interviews could be compared and verified using website statistics/flow tools. Because of software limitations in the current version of the DAC, it was not possible to install tools such as these for this study.

### Conclusion

Overall, this study shows that usability and usefulness of the researched portal are well appreciated. The use of an online portal seems a feasible option for providing eHealth to patients with dementia and their carers. It shows that (beginning) dementia or older age do not have to be a hindrance to computer or Internet use, although cognitive abilities change with dementia and are likely to affect computer use (eg, working memory, perceptual speed). Good website design can help to deal with these dementia-related changes. Using the correct font, colors, writing style, and navigation layout can make websites easier for people with dementia and their (often-older) carers to access. Designing websites in close collaboration with the target group and usability and usefulness testing within this group warrants optimal design and use of patient portals. Based on findings from our study, and on earlier research, we made several practical suggestions for good website design, which can be useful for others intending to design an online portal for people with dementia and their carers. It is recommended to improve on usability issues as soon as possible. For example, problems with finding the URL could be alleviated by adding redirects on more URLs (eg, accessing websites easier for people with dementia and their [often-older] carers). Because this is a very critical step—it is the first contact with the portal—it is highly relevant to find ways to fix this, such as by allowing log-in information to be saved or by considering other means of logging in. For other practical tips on portal design, please refer to the Textbox 1.

**Practical tips for portal design.**

**Clearly identify clickable targets.** Participants clicked even when not necessary, making it necessary to ensure that they do not click anything by accident to avoid confusion.

**Break information into short sections.** Long texts were found to be confusing to some participants; they found it hard to “follow the text.”

**Make use of the “recognize, rather than recall” principle.** Users appreciated that they could quickly recognize that the site was part of the Alzheimer Center because it used the “theme” colors.

**Minimize complex steps such as logging in.** Both the main log-in and the log-in required for further personal file access were considered too complex for users.

## Appendix

Multimedia Appendix 1 Questions of the online survey.

Multimedia Appendix 2 Survey participant flowchart.

## Abbreviations

DAC: Digital Alzheimer Center
EHR: electronic health record
SUS: System Usability Scale
USE: User Satisfaction and Ease of use
