# Supplementary material for: Evaluation of the Digital Alzheimer Center: Testing Usability and Usefulness of an Online Portal for Patients with Dementia and Their Carers
Source: JMIR Res Protoc. 2016 Jul 21;5(3):e144. doi: 10.2196/resprot.5040 (PMC4974452; doi:10.2196/resprot.5040)
Supplement: Multimedia Appendix 1 [file resprot_v5i3e144_app1.pdf]

Survey vragen

Timing

These page timer metrics will not be displayed to the recipient.

First Click: *0 seconds.*

Last Click: *0 seconds.*

Page Submit: *0 seconds.*

Click Count: *0 clicks.*

Geachte heer, mevrouw,

Hartelijk dank dat u bereid bent deze vragenlijst in te vullen.

Als bezoeker van het VUmc Alzheimercentrum heeft u toegang tot het Digitaal Alzheimercentrum, het DAC. Wij willen het DAC graag zo goed mogelijk laten aansluiten bij de wensen en behoeften van de gebruikers. Wij vinden het daarom belangrijk te onderzoeken wat de ervaringen van onze gebruikers zijn met het DAC. In het eerste deel van deze vragenlijst willen we u vragen uw waardering te geven over de huidige functies van het DAC. In het tweede deel willen we u vragen uw mening te geven over toekomstige mogelijkheden voor het DAC.

Het invullen van de gehele vragenlijst duurt ongeveer 10 minuten.

U kunt op de knop rechtsonder ("Verder") klikken om verder te gaan met de vragenlijst.

Afbeelding: Voorbeeld van de startpagina van het DAC.

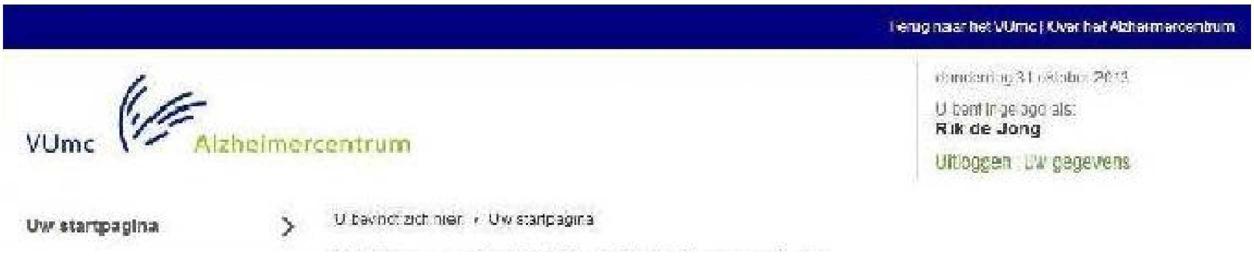

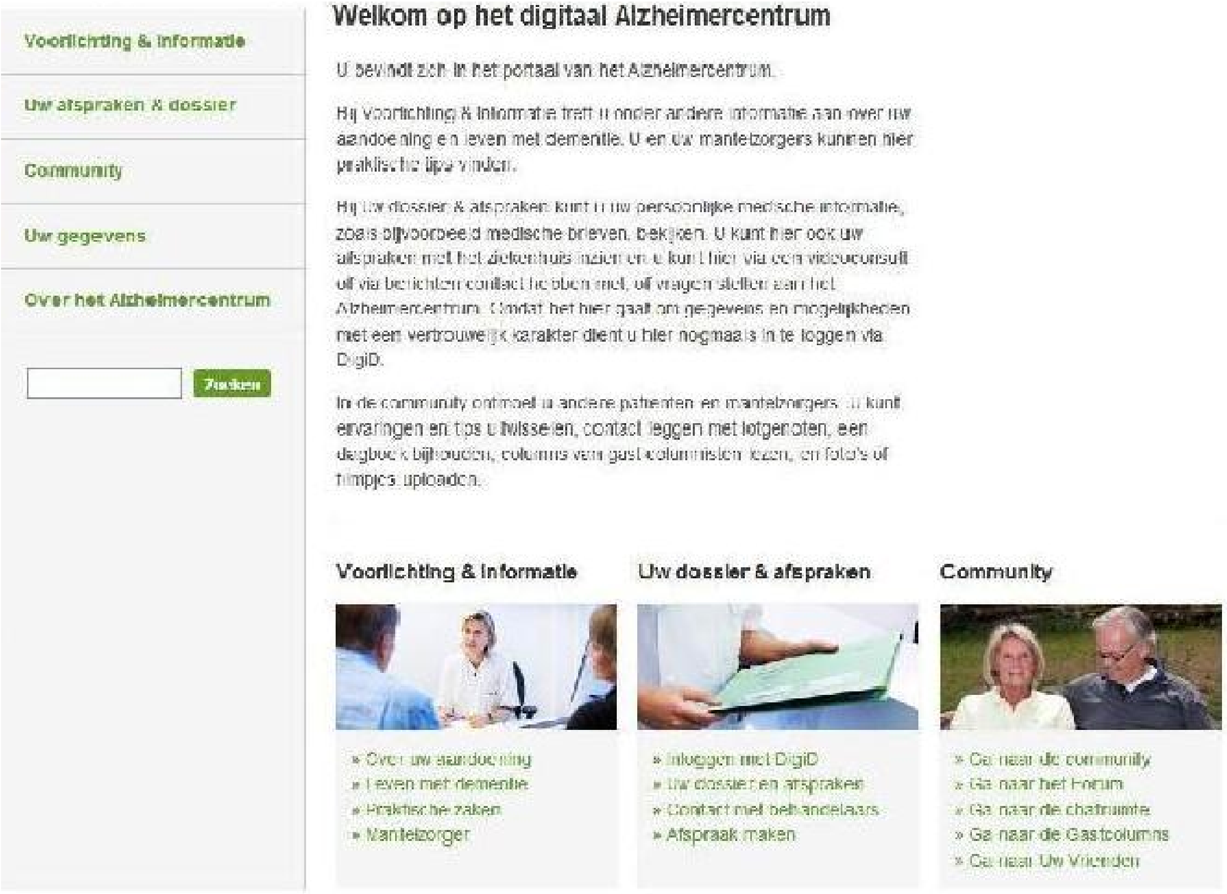

VUmc Alzheimercentrum

Wat is uw leeftijd?

Hoe bent u betrokken bij het VUmc Alzheimercentrum?

- ○ Ik ben een mantelzorger
- ○ Ik ben patiënt

Hoeveel ervaring heeft u met computergebruik?

- ○ Geen
- ○ Weinig
- ○ Matig
- ○ Veel
- ○ Heel veel

Heeft u het Digitaal Alzheimer Centrum, het DAC, wel eens gebruikt?

- Ja
- Nee

Waarom heeft u het DAC niet gebruikt?

Hoe vaak gebruikt u het DAC?

- Minder dan eens per maand
- Maandelijks
- Wekelijks
- Dagelijks
- Ik heb het één keer gebruikt, maar daarna niet meer

Waarom gebruikt u het DAC niet meer?

Uw waardering van het DAC

Hoe gemakkelijk is het om op het DAC de informatie te vinden die u zoekt?

- Heel gemakkelijk
- Gemakkelijk
- Neutraal
- Moeilijk
- Heel moeilijk

Hoe gemakkelijk is het om te leren omgaan met het DAC?

- Heel gemakkelijk
- Gemakkelijk
- Neutraal
- Moeilijk
- Heel moeilijk

Hoe duidelijk vindt u de indeling van de verschillende onderdelen van het DAC?

- Heel duidelijk
- Duidelijk
- Neutraal
- Onduidelijk
- Heel onduidelijk

Zijn de teksten in voor u begrijpelijke taal geschreven?

- Heel begrijpelijk
- Begrijpelijk
- Neutraal
- Onbegrijpelijk
- Heel onbegrijpelijk

Hoe duidelijk is het welke informatie u bij welk onderdeel kunt vinden?

- Heel duidelijk
- Duidelijk
- Neutraal
- Onduidelijk
- Heel onduidelijk

Hebben de illustraties een toegevoegde waarde?

- Ja, heel veel
- Ja
- Neutraal
- Nee
- Nee, helemaal niet
- Weet ik niet / ik heb nooit op de afbeeldingen gelet

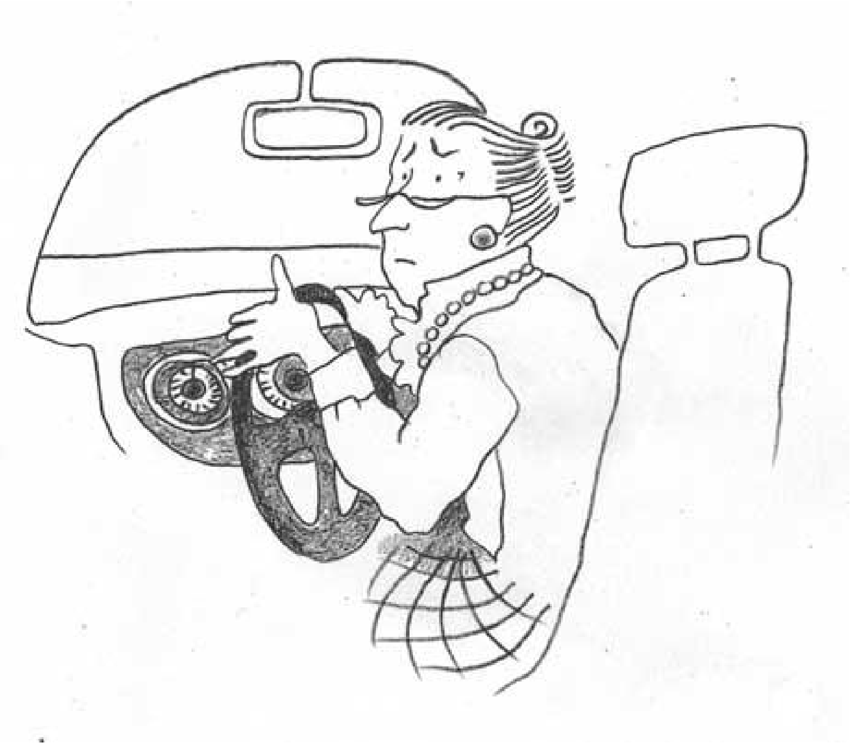

Heeft u video's bekeken op het DAC?

- Ja
- Nee

Hoe beoordeelt u de video's op:

	Heel goed	Goed	Neutraal	Slecht	Heel slecht
Duidelijkheid	○	○	○	○	○

[illegible]

Leerzaamheid	○	○	○	○	○
Begrijpelijkheid	○	○	○	○	○
Snelheid	○	○	○	○	○

Kunt u dit toelichten?

[ ]

Vindt u het kleurgebruik op het DAC prettig?

- ○ Ja
- ○ Neutraal
- ○ Nee

Waarom vindt u het kleurgebruik niet prettig?

(U kunt hier meerdere antwoorden aanvinken)

- ☐ De kleuren zijn te donker
- ☐ De kleuren zijn te licht
- ☐ Ik vind de kleuren niet mooi
- ☐ Ik vind de kleuren niet duidelijk

Vindt u het lettertype dat op het DAC gebruikt wordt prettig?

- ○ Ja
- ○ Neutraal
- ○ Nee

Waarom vindt u het lettertype niet prettig?

- ☐ De letters zijn te klein
- ☐ De letters zijn te groot
- ☐ De letters zijn niet duidelijk

De letters zijn niet duidelijk

We hebben nu een aantal kenmerken van het DAC besproken. Misschien zijn er voor u belangrijke aspecten die niet besproken zijn. Heeft u andere suggesties om het DAC gebruiksvriendelijker te maken?

Kunt u van de de hieronder weergegeven onderdelen aangeven of u die informatie heeft gelezen?

	Ik heb dit gelezen	Ik heb dit niet gelezen
Informatie over uw aandoening	○	○
Informatie over leven met dementie	○	○
Informatie voor mantelzorgers	○	○
Gastcolumns	○	○
Informatie over het Alzheimercentrum	○	○

Kunt u van de hieronder weergegeven onderdelen aangeven of u die gebruikt hebt?

	Ik heb dit gebruikt	Ik heb dit niet gebruikt	Ik heb dit geprobeerd, maar dit lukte niet
Afspraken en dossier: uw afspraken	○	○	○
Afspraken en dossier: E-consult	○	○	○
Afspraken en dossier: Correspondentie (uw brieven)	○	○	○
[illegible]			

Afspraken en dossier: VUmc gegevens	○	○	○
Afspraken en dossier: contact met de hulpverlener	○	○	○

Hoe ervaart u het inloggen met Digid?

- ○ Heel gemakkelijk
- ○ Gemakkelijk
- ○ Neutraal
- ○ Moeilijk
- ○ Heel moeilijk

Heeft u "Het forum" wel eens gebruikt?

- ○ Ja
- ○ Nee

Wat heeft u gedaan op het forum?

(U kunt hier meerdere antwoorden aanvinken)

- ☐ Ik heb berichten gelezen
- ☐ Ik heb een bericht geplaatst
- ☐ Ik heb een antwoord op een bericht geplaatst

Heeft u de "Foto- en videogalerie" wel eens gebruikt?

- ○ Ja
- ○ Nee

Heeft u de "Chatruimte" wel eens gebruikt?

- ○ Ja

Nee

Heeft u het "Online-dagboek" wel eens gebruikt?

Ja

Nee

Heeft u de functie "Uw vrienden" wel eens gebruikt?

Ja

Nee

Welke mogelijkheid bij 'uw vrienden' heeft u gebruikt?

(U kunt hier meerdere antwoorden aanvinken)

Ik heb naar vrienden gezocht

Ik heb vriendschapsverzoeken verstuurd

Ik heb vriendschapsverzoeken ontvangen

Ik heb berichten gestuurd naar vrienden

Heeft u de functie "Uw gegevens" wel eens gebruikt?

Ja

Nee

Welke mogelijkheid bij 'uw gegevens' heeft u gebruikt?

(U kunt hier meerdere antwoorden aanvinken)

Ik heb mijn gegevens gewijzigd

Ik heb aangegeven dat anderen mij mogen vinden

Ik heb mijn interesses ingevuld

Hoe nuttig vindt u het DAC?

- Heel nuttig
- Nuttig
- Neutraal
- Niet nuttig
- Helemaal niet nuttig

Heeft het DAC voor u toegevoegde waarde, boven de gebruikelijke zorg vanuit het VUmc Alzheimercentrum?

- Heel veel
- Weinig
- Geen
- Weet ik niet

Vindt u dat u met het DAC gemakkelijker contact met uw zorgverleners kunt opnemen?

- Ja, veel gemakkelijker
- Ja, gemakkelijker
- Neutraal
- Nee, moeilijker
- Nee, veel moeilijker

Maakt het DAC het u gemakkelijker uw gegevens te wijzigen of te beheren?

- Ja, veel gemakkelijker
- Ja, gemakkelijker
- Neutraal
- Nee, moeilijker
- Nee, veel moeilijker

Helpt het DAC u bij het begrijpen van dementie?

- Ja, heel veel

- Ja, een beetje
- Neutraal
- Nee, niet echt
- Nee, helemaal niet

Helpt het DAC u in het omgaan met dementie?

- Ja, heel veel
- Ja, een beetje
- Neutraal
- Nee, niet echt
- Nee, helemaal niet

Helpt het DAC u om informatie te vinden die u nodig heeft?

- Ja, heel veel
- Ja, een beetje
- Neutraal
- Nee, niet echt
- Nee, helemaal niet

Zou u het DAC aan anderen aanraden?

- Ja
- Misschien
- Nee

Kunt u dit toelichten?

Nu volgt een aantal mogelijke nieuwe functies voor het DAC. Kunt u per functie aangeven of u die functie zou gebruiken? Klik rechtsonder op 'verder' om door te gaan.

Hoewel u het DAC niet gebruikt, zouden wij alsnog graag willen weten of u de volgende nieuwe functies zou willen gebruiken.

Zou u gebruik maken van een openingspagina, waarop u in één oogopslag een overzicht krijgt van nieuwe gebeurtenissen op het DAC?

- Ja
- Misschien
- Nee

Kunt u dit toelichten?

Zou u meer informatie over uzelf op het DAC willen plaatsen? (Zoals bijvoorbeeld een foto.)

- Ja
- Misschien
- Nee

Kunt u dit toelichten?

Zou u gebruik maken van een apart deel in het forum, waar u uzelf kunt voorstellen?

- Ja
- Misschien
- Nee

Kunt u dit toelichten?

Zou u gebruik maken van de mogelijkheid op afstand contact met uw arts te maken, via een video-verbinding?

- Ja

- Misschien
- Nee

Kunt u dit toelichten?

Zou u een video-opname van uw uitslaggesprek bekijken, om dit gesprek later nog eens terug te kunnen zien?

- Ja
- Misschien
- Nee

Kunt u dit toelichten?

U heeft aangegeven dat u wel eens heeft geprobeerd een functie onder "Uw afspraken en dossier" te gebruiken, maar dat dat niet lukte. Als u hieronder uw email-adres invult, neemt iemand contact met u op om u hierbij te helpen.

Het antwoord op deze vraag zal in verdere analyses verwijderd worden. Uw gegevens blijven dus anoniem bewaard.

Block 1
