# Supplementary figures and images for: Evaluation of the Digital Alzheimer Center: Testing Usability and Usefulness of an Online Portal for Patients with Dementia and Their Carers
Source: JMIR Res Protoc. 2016 Jul 21;5(3):e144. doi: 10.2196/resprot.5040 (PMC4974452; doi:10.2196/resprot.5040)

## Appendix B. Flowchart of survey participants.

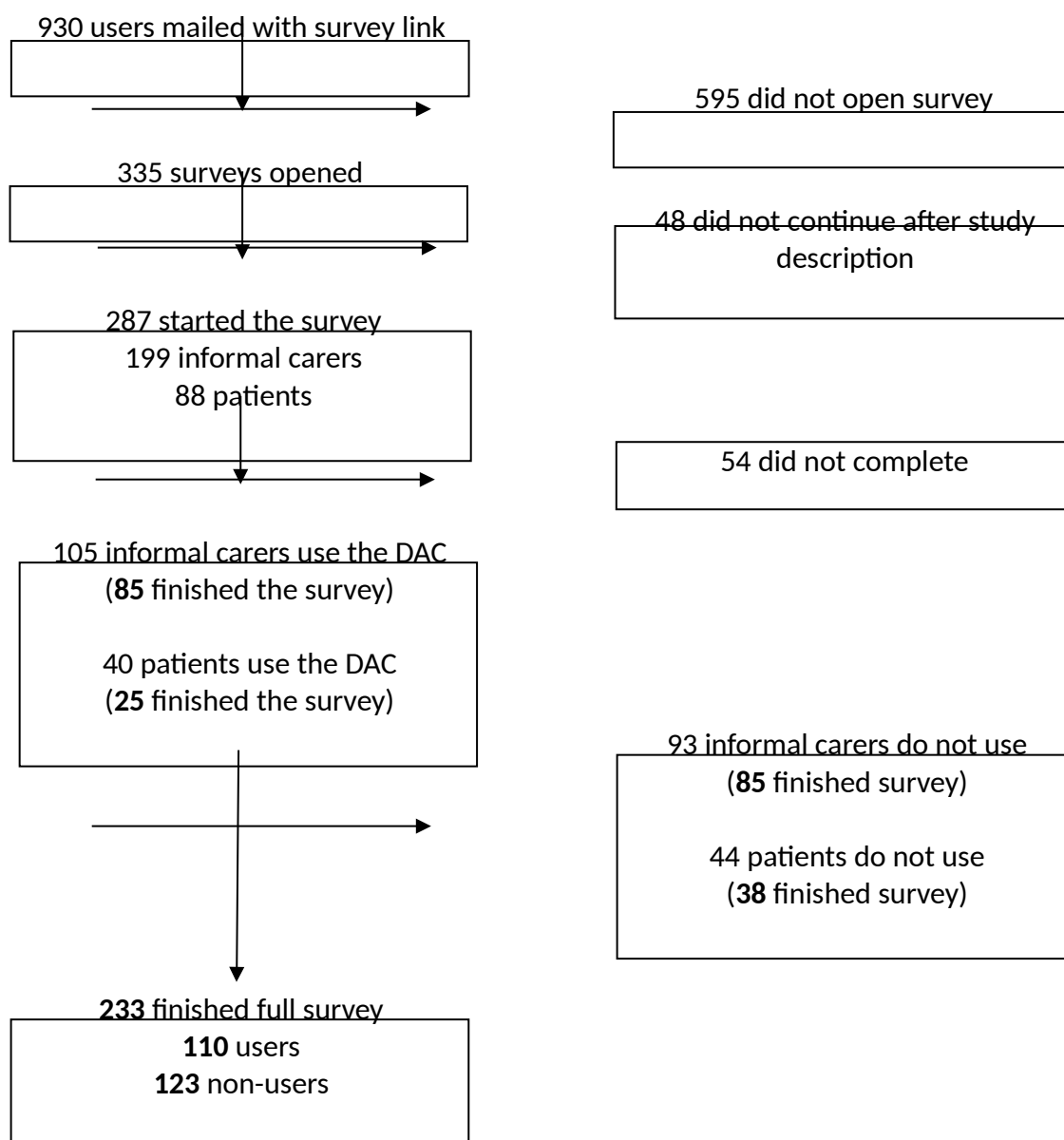

Supplement: Multimedia Appendix 2 [file resprot_v5i3e144_app2.pdf]
